# A new classifier-based strategy for *in-silico* ion-channel cardiac drug safety assessment

**DOI:** 10.3389/fphar.2015.00059

**Published:** 2015-03-24

**Authors:** Hitesh B. Mistry, Mark R. Davies, Giovanni Y. Di Veroli

**Affiliations:** ^1^Manchester Pharmacy School, University of ManchesterManchester, UK; ^2^QT-Informatics LimitedMacclesfield, UK; ^3^Cancer Research UK Cambridge Institute, University of CambridgeCambridge, UK

**Keywords:** cardiac toxicity, mathematical model, ion-channel pharmacology, predictive pharmacology, biostatistics

## Abstract

There is currently a strong interest in using high-throughput *in-vitro* ion-channel screening data to make predictions regarding the cardiac toxicity potential of a new compound in both animal and human studies. A recent FDA think tank encourages the use of biophysical mathematical models of cardiac myocytes for this prediction task. However, it remains unclear whether this approach is the most appropriate. Here we examine five literature data-sets that have been used to support the use of four different biophysical models and one statistical model for predicting cardiac toxicity in numerous species using various endpoints. We propose a simple model that represents the balance between repolarisation and depolarisation forces and compare the predictive power of the model against the original results (leave-one-out cross-validation). Our model showed equivalent performance when compared to the four biophysical models and one statistical model. We therefore conclude that this approach should be further investigated in the context of early cardiac safety screening when *in-vitro* potency data is generated.

## Introduction

The consequence of a drug carrying cardiac liability is a significant cause of concern to the Pharmaceutical industry. In the late 1980s to the early 1990s, a number of significant withdrawals from market occurred due to the detection of drug induced cardiac arrhythmias, e.g., Torsades de Pointes (ventricular tachycardia) such as prenylamine, lidoflazine, and terodiline from UK and EU market (Stockbridge et al., 2013). The exact number of drugs that cause Torsades de Pointes is unknown, however the CredibleMeds initiative (www.crediblemeds.org) has cataloged 42 drugs with a “Known Risk of Torsades de Pointes.” These arrhythmias were mostly attributed to drugs causing significant prolongation to the QTc interval (time taken for ventricular depolarisation and repolaristaion), which is in turn related to the delay in repolarisation of the ventricular wall. Causative link was then established with drug blocking of the hERG (*human-ether-a-go-go*) ion-channel within ventricular myocytes (Pollard et al., 2010). As a consequence, routine screening of compounds via *in-vitro* high-throughput screening (HTS) devices was quickly incorporated into early stages of drug-development (Pollard et al., 2010). Initial screens focused only on the hERG channel but in recent years it has become apparent that other ion-channels might critically affect cardiac electrophysiology. In particular, hCav1.2 and hNav1.5 have been recognized as key depolarising ion channels with important roles in the mechanisms causing arrhythmia, e.g., long QT syndromes LQT3 and LQT8 syndromes (Lehnart et al., 2007). Therefore, screenings have now also been extended to include these other ion-channels (Cavero and Holzgrefe, 2014).

Research efforts have highlighted the potential use of *in-silico* biophysical models (Trayanova, 2011) of cardiac myocytes to predict the cardiac risk *in-vivo* or even in the clinical setting, based on *in-vitro* ion-channel screening data (Cavero and Holzgrefe, 2014). These models describe the dynamic opening and closing of ion-channels and resulting temporal variation of cell Action Potential (AP) via a set of differential equations. They are parameterized based on experimental data from electrophysiological recordings of isolated ion-channels and also whole cell AP recordings. Their goal is generally to produce a descriptive model of the cardiac myocyte which is then used to better understand general cardiac biology. Nevertheless, there is still uncertainty as to which model is best suited to assist in cardiotoxicity prediction.

Answering these questions is in line with recent initiatives from a FDA sponsored think tank suggesting the usage of *in-silico* tools in correlating non-clinical *in-vitro* studies with proarrhythmic risk (Sager et al., 2014). Two techniques have been used in the literature: (1) biophysical models which describe the dynamics of a cardiac myocyte through differential equations of which there are 4 examples (Mirams et al., 2011, 2014; Davies et al., 2012; Beattie et al., 2013) and (2) statistical models which focus on known ion-channel pharmacology of which there is only one literature example (Kramer et al., 2013). Here we investigate an alternative strategy based on a one-equation classifier model and show that similar predictive power can be obtained with this strategy. In particular, we highlight the capacity of such a model in handling all datasets, in contrast with the original studies where a specific model was used at each time. The models predictive power within each data-set under consideration is also assessed via a leave-one-out cross validation exercise where an optimal parameter set for each data-set is used. We will then discuss the advantages of this alternative approach.

## Materials and methods

### Data-sets

All data-sets are reported in the Supplementary Material. Here we present a brief summary:

- **Human 1** (Kramer et al., 2013) contains 55 compounds and assessed the Torsades de Pointes risk of each compound. All ion-channel potency data was generated from two *in-vitro* HTS platforms, Qpatch, and PatchXpress. The cell lines used were HEK293 and CHO (Chinese Hamster Ovary). The *in-silico* model used within that study was a statistical (logistic regression) model which classified a compound as posing a Torsades de Pointes risk or not.
- **Human 2** (Mirams et al., 2011) contains 31 compounds and assessed the Torsades de Pointes risk of each compound. Ion-channel potency data was derived from numerous literature *in-vitro* reports. The *in-silico* model used within that study was a biophysical model (39 differential equations) which classified compounds into one of four Torsades de Pointes risk categories. This was then simplified to a binary classification question of whether a compound posed a Torsades de Pointes risk or not.
- **Human 3** (Mirams et al., 2014) contains 34 compounds and assessed the QTc prolongation potential of each compound. We investigated the data-set which gave the authors the best result. This contained *in-vitro* hERG manual patch-clamp data (obtained from regulatory documents for each compound) and *in-vitro* HTS data for the other ion-channels (IonWorks Quattro using CHO cells). The *in-silico* model used within that study was a biophysical model (41 differential equations) which classified compounds as QTc prolongers or not at the mean drug concentration. QTc prolongation was defined by a mean increase in QTc interval of at least 5 ms.
- **Dog** (Davies et al., 2012) contains 53 compounds and assessed the time taken for the canine AP duration to repolarise by 90% (APD_90_) within the canine AP *in-vitro* assay. All ion-channel potency data was generated from the same *in-vitro* HTS platform, IonWorks\ FLIPR with CHO cells. The *in-silico* model used within that study was a biophysical model (29 differential equations) which classified compounds to one of three categories: prolongation, shortening or no effect. Prolongation/shortening was defined by a greater than 10% change in either direction compared to control at a given drug concentration.
- **Rabbit** (Beattie et al., 2013) contains numerous data-sets with potency estimates generated from different platforms (*in-vitro* and *in-silico* i.e., computational chemistry predicted potency estimates) and assessed the change in QTc within the *ex-vivo* rabbit ventricular wedge assay. Here we considered the dataset that gave the authors the best result there. This contained a total of 77 compounds where all the ion-channel potency data was generated from the same *in-vitro* HTS platform, PatchXpress with HEK293 and CHO cells. The *in-silico* model used within that study was a biophysical model (45 differential equations) which classified compounds in the same way as the above dog study.

### Drug effect

The ion-channel screening data extracted from the articles was converted into block or agonism of an ion-channel at a given drug concentration of interest in the following way. We first defined the effect *S* of a drug on ion-channel *x* as:

$$S_{x}=1-\frac{1}{1+{\left(\frac{IC_{50}}{\left[D\right]}\right)}^{n}},$$

for an inhibitor and

$$S_{x}=1+\frac{E_{\max}}{1+{\left(\frac{EC_{50}}{\left[D\right]}\right)}^{n}},$$

for an agonist, where *x* = *I*_*Kr*_, *x* = *I*_*CaL*_ or *x* = *I*_*Na*_ refer to the hERG, hCav1.2, and hNav 1.5 ion-channels respectively, *[D]* is the concentration of drug, *IC*_50_ is the amount of drug required to reduce the effect by 50%, *EC*_50_ is the amount of drug required to increase the effect by *E*_*max*_/*2*, *E_max_* the maximum effect of an agonist and *n* the hill coefficient. The *IC*_50_, *EC*_50_, *E_max_*, and *n* values were taken as per the published data-sets. There, drug effects within bio-physical models were modeled by scaling conductances of ion-channels by *S_x_* which was also done here. Only effects against the above three ion-channels were considered as they were consistently measured across all studies.

### One-equation classifier model

It is well understood that the hERG channel is responsible for repolarisation and that hCav1.2 and hNav1.5 ion-channels are largely responsible for depolarisation of the action potential. We hypothesized that the balance between the block/agonism of depolarisation and repolarisation forces is the main parameter driving drug effects' predictions. The following classifier was suggested:

$$Z=\frac{1+a_{0}S_{CaL}+a_{1}S_{Na}}{1+a_{2}S_{Kr}}$$

where *a*_0_, *a*_1_, and *a*_2_ are coefficients that need to be determined. For simplicity, we initially chose to set the coefficients in the model to 1, as the first goal of this study was to assess the discriminatory value of the model without specific parameters optimization.

### ROC curve

The model was initially assessed through a receiver operating characteristic (ROC) curve analysis (Zou et al., 2007). The ROC curve is a plot of the sensitivity (true positive rate) as a function of 1—specificity (i.e., false positive rate) for different cut-off values of a discriminant variable. The ROC curve can be assessed by looking at the obtained area under the curve (AUC). A value equal to 1 implies 100% sensitivity/specificity while a value equal to 0.5 represents 50% sensitivity/specificity. The ROC curve can also be inspected visually by comparing it to the line of unity: the further the curve is above this line the better.

Within this analysis, the following binary classification questions were asked:

1. For the dog and rabbit data-sets:
  - Does a compound cause prolongation?
  - Does a compound cause shortening?
2. For human 1 and 2 data-sets, does a compound have a Torsades de Pointes risk or not [definitions as per original articles (Mirams et al., 2011; Kramer et al., 2013)]?
3. For human 3 data-set, does a compound cause QTc prolongation?

For the animal data-sets the above setup does mean a compound can be classified as both prolongation and shortening, however this is relaxed when considering the predictivity of the model as described below.

### Leave-one-out cross validation

After assessing the structural models discriminatory value through a ROC analysis we assessed the predictive power of the model via a leave-one-out cross validation. We used *n* − 1 compounds to train the model and the *n*th compound to test it. This process was repeated until all n compounds were used as a test-set. Here the model parameter values, *a*_0_, *a*_1_, and *a*_2_, were optimized for each data-set (see Supplementary Material). Compounds within the animal data-sets were classified as causing prolongation, no-effect or shortening. The result of this analysis was summarized through sensitivity, specificity and balanced accuracy [i.e., (sensitivity + specificity)/2] metrics. These metrics were then compared to the reported results of the original articles model predictions.

## Results

### ROC analysis and model comparison

**Figure 1:**
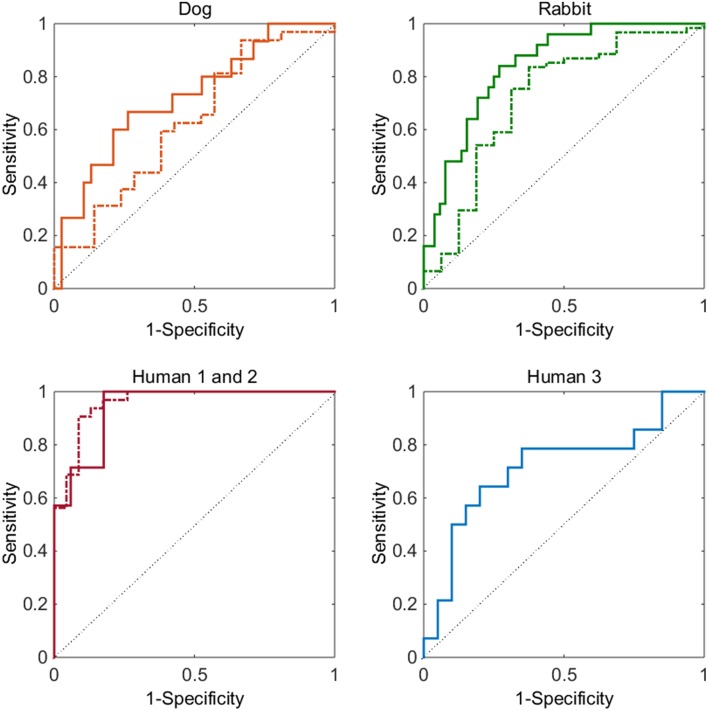
**Top left graph, dog data-set: ROC curve for prolongation (solid line) and for shortening (dashed line)**. Top right graph, rabbit data-set: ROC curve for prolongation (solid line) and for shortening (dashed line). Bottom left graph: ROC curve for Torsades de Pointes risk for the human 1 (dashed) and human 2 (solid) data-sets. Bottom right graph: ROC curve for QTc prolongation for the human 3 data-set (solid). In all graphs the thin solid line represents a ROC curve of random chance.

Overall it can be seen (Figure 1) that the ROC curve for the five cases investigated sit comfortably above the identity line. This shows that the model demonstrates good discriminatory power for all datasets without data-set specific parameter optimization. The strongest result can be seen for assessing whether a compound has a Torsades de Pointes risk or not (bottom left graph of Figure 1). The AUC values of the ROC curves for human 1 (dashed line) and 2 (solid line) data-sets are 0.94 and 0.96, respectively. These values are considered to be excellent and are close to a perfect classifier. The signal for QTc prolongation was also found to be good (ROC AUC of 0.72). The model also performed well for the animal data-sets: the ROC AUC values for dog and rabbit prolongation were 0.72 and 0.84 respectively, while the AUC values for shortening were 0.63 and 0.72 respectively.

We then performed a leave-one-out cross validation where model parameters were allowed to vary and an optimum set derived for each data-set. The results of this additional analysis and comparison of our model's prediction to the reported predictions can be seen in Table 1. Our model results were overall comparable to the published models' results. Sensitivity was increased in all data-sets except the Human 1 and 2 data-sets where the difference was minimal. Specificity was in good agreement against all the literature models. The balanced accuracy was either equal or improved over the current published models except in the Human 2 data-set where our model performed slightly worse than the biophysical model used there.

**Table 1:**
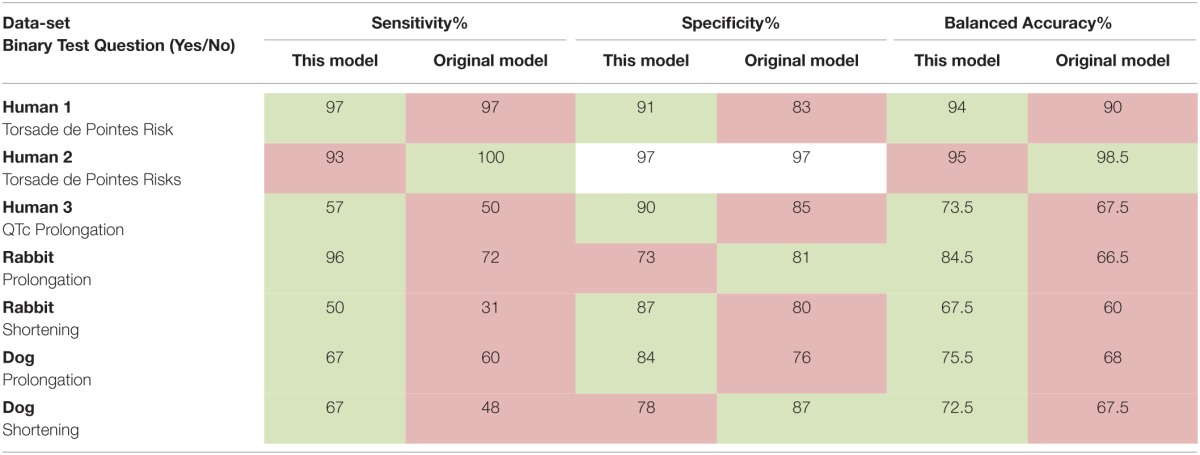
**Sensitivity, specificity, and balanced accuracy of our model compared with the original models' predictions**.

*Better metrics are highlighted in green. Worse metrics are highlighted in red*.

## Discussion

There is currently strong interest from both regulatory agencies and pharmaceutical companies in the use of *in-silico* models of cardiac myocytes as a tool for integrating the cardiac ion-channel liability of new compounds. This interest is motivated by the question of predicting the effects of a new compound's cardiac risk in animal or man simply by using *in-vitro* ion-channel potency data (Cavero and Holzgrefe, 2014). This question has been predominantly answered to date by either empirically defining cut-off thresholds for *IC*_50_ values or by using *in-silico* approaches with biophysical models (Mirams et al., 2011, 2014; Davies et al., 2012; Beattie et al., 2013). Only one statistical model approach have been used to date to our knowledge (Kramer et al., 2013). Biophysical models benefit by incorporating representations of cardiac myocyte biology that may provide an intuitive leap for the experienced electrophysiologist to articulate depolarization mechanisms. Indeed, there are now many models representing different cell types and species available (see for instance the CellML database http://models.cellml.org/cellml).

Nevertheless, these biophysical models can suffer from their size and display complex behavior which can be highly parameter-sensitive (Gutenkunst et al., 2007). Their formulations vary greatly between species and cell-type and there are also competing formulations for the same species and cell-type. It is difficult to fully validate such models in practice. Moreover, this pre-existing complexity can be compounded by the additional incorporation of other major biological processes such as drug binding kinetics, regulatory signaling or spatio-temporal effects as well as statistical assessment. Thus, we wanted to explore how a model that minimizes mathematical formulation to describe AP prolongation compares to biophysical models in its predictive capacity. Such a tool, which is far less complex than current biophysical models, could facilitate the additional incorporation of other fundamental processes (Lu et al., 2013) and of statistical considerations (Chain et al., 2011).

We introduced a new classifier-type model, which is relatively simple (one equation) and can be directly interpreted in terms of the balance between depolarisation and repolarisation forces. The model performance was assessed via a ROC analysis and a leave-one-out cross validation across various literature data-sets. When comparing this model performance against published biophysical models, we found comparable results (same scoring system as per original studies was used). As a consequence, these results suggest that biophysical models might not provide significant benefit over the approach taken here to describe the correlation between ion-channel block and compound risk. It is possible that these models may be better suited to understand and articulate the cellular and tissue processes mechanistically, without having necessarily reached the required maturity that would imply added benefit in terms of predictive pharmacology.

With our new approach, other biological processes can be also incorporated by emphasizing on their influence rather than their precise mathematical description. The influence of other ion channels such as I_k1_, I_NaCa_, I_to_ or I_Ks_ can be incorporated in similar ways as per the ion-channels considered here. We can also imagine the possibility of accounting for slow vs. fast ion-channel block with additional parameters. New approaches have indeed emerged recently to elucidate drug binding dynamics using modern HTS assays (Di Veroli et al., 2013, 2014). Other processes such as spatio-temporal effects or regulatory processes can be integrated as separated factors and could also account for variability across populations. Furthermore, a simple model like this is easier to implement for scientists of all backgrounds and does not require extensive amount of domain specific knowledge. It can potentially lead to both cost and time-savings for safety pharmacology groups who are interested in assessing ion-channel related cardiac toxicity risk of new compounds.

All mathematical models, either simple or complex, might be followed later on by experiments to test for cardiac safety screening. Nevertheless, they represent a valuable tool for assessing risk and prioritizing new compounds at early stages. Biophysical models explicitly describe the mechanisms behind AP propagation and other important biological processes and are thus invaluable for gaining qualitative understanding of biological processes. Although our simple model does not claim to provide the level of description given by biophysical models, our results showed similar predictive power when correlating ion-channel block with AP or QT prolongation/shortening using published datasets. At the same time, our model was applied across different species and also avoided model complexity and its inherent risks. Thus, we propose this approach to form the basis of data-driven systems pharmacology strategies as it simplifies the incorporation of other aspects such as uncertainty and biological variability. Being able to include uncertainty is indeed a key hallmark of simple models compared to larger models as it is easier to place such model within a statistical framework. We hope that these surprising but significant results indicate how different model strategies can be used inclusively and can help the researcher in understanding the limitations in prediction over different modeling scales.

### Conflict of interest statement

The authors declare that the research was conducted in the absence of any commercial or financial relationships that could be construed as a potential conflict of interest.
