# Supplementary material for: A new classifier-based strategy for in-silico ion-channel cardiac drug safety assessment
Source: Front Pharmacol. 2015 Mar 24;6:59. doi: 10.3389/fphar.2015.00059 (PMC4371651; doi:10.3389/fphar.2015.00059)
Supplement: Supplementary file 1 [file DataSheet1.DOCX]

***Supplementary Material***

**A new classifier-based strategy for in-silico ion-channel cardiac drug safety assessment**

**Hitesh B. Mistry PhD,^1*^ Mark R. Davies PhD,^2^ Giovanni Y. Di Veroli PhD ^3^**

^1^Manchester Pharmacy School, University of Manchester, Manchester, M13 9PL, UK

^2^QT-Informatics Limited, UK

^3^CRUK Cambridge Institute, University of Cambridge, UK

*** Correspondence:** Hitesh B. Mistry, Manchester Pharmacy School, University of Manchester, Manchester, M13 9PL, UK hitesh.mistry@manchester.ac.uk

1. **Supplementary Tables**

For each data-set, the in-vitro data (amount of ion-channel block or agonism) is reported. We also report the data-set specific outcome for the in-vivo end-points considered in each-one of the studies and our predictions of these end-points based on the in-vitro data following the leave-one-out cross-validation (LOOCV).

Human 1 data-set

The Response column in this data-set corresponds to whether a compound carries a Torsades de Pointes risk (1) or not (0).

| Compound | SKr | Sna | SCaL | Response | LOOCV |
| --- | --- | --- | --- | --- | --- |
| Amiodarone | 1.00 | 1.00 | 1.00 | 1 | 0 |
| Astemizole | 0.93 | 1.00 | 1.00 | 1 | 1 |
| Bepridil | 0.82 | 0.99 | 0.97 | 1 | 1 |
| Ceftriaxone | 0.95 | 0.96 | 0.87 | 0 | 0 |
| Chlorpromazine | 0.98 | 0.99 | 0.99 | 1 | 1 |
| Cilostazol | 0.99 | 1.00 | 1.00 | 1 | 1 |
| Cisparide | 0.87 | 1.00 | 1.00 | 1 | 1 |
| Clozapine | 0.97 | 1.00 | 0.98 | 1 | 1 |
| Dasatnib | 1.00 | 1.00 | 1.00 | 0 | 0 |
| Diazepam | 1.00 | 1.00 | 1.00 | 0 | 0 |
| Diltiazem | 0.99 | 0.99 | 0.86 | 0 | 0 |
| Disopyramidie | 0.95 | 1.00 | 1.00 | 1 | 1 |
| Dofetilide | 0.94 | 1.00 | 1.00 | 1 | 1 |
| Donepezil | 1.00 | 1.00 | 1.00 | 0 | 1 |
| Droperidol | 0.79 | 1.00 | 1.00 | 1 | 1 |
| Duloxetine | 1.00 | 1.00 | 0.99 | 0 | 0 |
| Flecainide | 0.67 | 0.89 | 0.97 | 1 | 1 |
| Halofrantine | 0.69 | 1.00 | 0.92 | 1 | 1 |
| Haloperidol | 0.91 | 1.00 | 1.00 | 1 | 1 |
| Ibutilide | 0.11 | 1.00 | 1.00 | 1 | 1 |
| Lamivudine | 0.99 | 0.99 | 0.74 | 0 | 0 |
| Linezolid | 0.95 | 0.98 | 0.64 | 0 | 0 |
| Loratidine | 1.00 | 1.00 | 1.00 | 0 | 0 |
| Methadone | 0.87 | 0.98 | 0.99 | 1 | 1 |
| Metronizadole | 0.88 | 0.92 | 0.49 | 0 | 0 |
| Mibefradil | 0.99 | 1.00 | 0.98 | 0 | 0 |
| Mitoxantrone | 1.00 | 1.00 | 0.99 | 0 | 0 |
| Moxifloxacin | 0.89 | 0.99 | 0.94 | 1 | 1 |
| Nifedipine | 1.00 | 1.00 | 0.60 | 0 | 0 |
| Nilotnib | 0.85 | 0.99 | 0.99 | 1 | 1 |
| Nitrendipine | 1.00 | 1.00 | 0.89 | 0 | 0 |
| Paliperidone | 0.92 | 1.00 | 1.00 | 1 | 1 |
| Paroxetine | 0.99 | 1.00 | 1.00 | 1 | 1 |
| Pentobarbital | 1.00 | 1.00 | 0.98 | 0 | 0 |
| Phenytoin | 0.97 | 0.94 | 0.83 | 0 | 0 |
| Pimozide | 0.99 | 1.00 | 1.00 | 1 | 1 |
| Piperacillin | 0.71 | 0.64 | 0.47 | 0 | 0 |
| Procainamide | 0.83 | 0.93 | 0.88 | 1 | 1 |
| Quinidine | 0.18 | 0.82 | 0.66 | 1 | 1 |
| Raltegravir | 0.99 | 0.99 | 0.97 | 0 | 0 |
| Ribavirin | 0.97 | 0.99 | 0.96 | 0 | 1 |
| Risperidone | 0.99 | 1.00 | 1.00 | 1 | 1 |
| Saquinavir | 0.99 | 0.99 | 0.94 | 0 | 0 |
| Sertindole | 0.94 | 1.00 | 1.00 | 1 | 1 |
| Sitagliptin | 1.00 | 1.00 | 1.00 | 0 | 0 |
| Solifenacin | 0.99 | 1.00 | 1.00 | 1 | 1 |
| Sotalol | 0.88 | 1.00 | 0.93 | 1 | 1 |
| Sparfloxacin | 0.93 | 1.00 | 0.98 | 1 | 1 |
| Sunitinib | 0.99 | 1.00 | 1.00 | 1 | 1 |
| Telbivudine | 0.96 | 0.98 | 0.97 | 0 | 1 |
| Terfenadine | 0.85 | 1.00 | 0.99 | 1 | 1 |
| Terodiline | 0.82 | 0.98 | 0.97 | 1 | 1 |
| Thioradizine | 0.34 | 0.59 | 0.78 | 1 | 1 |
| Verapamil | 0.74 | 1.00 | 0.69 | 0 | 1 |
| Voricanazole | 0.98 | 1.00 | 0.98 | 1 | 1 |

Human 2 data-set

The Response column in this data-set corresponds to whether a compound carries a Torsades de Pointes risk (1) or not (0).

| Compound | SKr | Sna | SCaL | Response | LOOCV |
| --- | --- | --- | --- | --- | --- |
| Ajmaline | 0.41 | 0.85 | 0.98 | 1 | 1 |
| Amiodarone | 0.98 | 1 | 1 | 1 | 1 |
| Amitriptyline | 0.99 | 1 | 1 | 0 | 0 |
| Bepridil | 0.5 | 0.99 | 0.86 | 1 | 1 |
| Cibenzoline | 0.96 | 0.89 | 0.97 | 0 | 0 |
| Desipramine | 0.93 | 0.93 | 0.94 | 0 | 0 |
| Diltiazem | 0.99 | 0.99 | 0.79 | 0 | 0 |
| Diphenhydramine | 0.99 | 1 | 1 | 0 | 0 |
| Dofetilide | 0.71 | 1 | 1 | 1 | 1 |
| Fluvoxamine | 0.89 | 0.99 | 0.93 | 0 | 1 |
| Haloperidol | 0.88 | 1 | 1 | 1 | 1 |
| Imipramine | 0.97 | 0.97 | 0.99 | 0 | 0 |
| Mexiletine | 0.92 | 0.91 | 0.96 | 0 | 0 |
| Mibefradil | 0.99 | 0.99 | 0.93 | 0 | 0 |
| Nifedipine | 1 | 1 | 0.89 | 0 | 0 |
| Nitrendipine | 1 | 1 | 0.1 | 0 | 0 |
| Phenytoin | 0.96 | 0.92 | 0.96 | 0 | 0 |
| Pimozide | 0.95 | 0.98 | 0.99 | 1 | 1 |
| Prenylamine | 0.79 | 0.99 | 0.99 | 1 | 1 |
| Propafenone | 0.65 | 0.83 | 0.88 | 0 | 1 |
| Propranolol | 0.99 | 0.99 | 1 | 0 | 0 |
| Quetiapine | 0.99 | 1 | 1 | 0 | 0 |
| Quinidine | 0.08 | 0.84 | 0.83 | 1 | 1 |
| Risperidone | 0.99 | 1 | 1 | 0 | 0 |
| Sertinadole | 0.9 | 1 | 1 | 1 | 1 |
| Terfenadine | 0.5 | 0.99 | 0.98 | 1 | 1 |
| Thioridazine | 0.03 | 0.65 | 0.57 | 1 | 1 |
| Verapamil | 0.64 | 1 | 0.55 | 0 | 1 |
| Chlorpromazine | 0.97 | 1 | 1 | 1 | 1 |
| Cisapride | 0.57 | 1 | 1 | 1 | 1 |
| Tedisamil | 0.97 | 0.99 | 1 | 1 | 1 |

Human 3 data-set

The Response column in this data-set corresponds to whether a compound carries a QTc prolongation risk (1) or not (0).

| Compound | SKr | Sna | SCaL | Response | LOOCV |
| --- | --- | --- | --- | --- | --- |
| Alfuzosin | 0.95 | 0.98 | 1.00 | 1 | 0 |
| Alvimopan | 0.98 | 0.99 | 0.99 | 0 | 0 |
| Ambrisentan | 0.88 | 0.97 | 0.98 | 1 | 0 |
| Darifenacin | 0.03 | 1.00 | 0.98 | 0 | 0 |
| Darunavir | 0.21 | 1.00 | 1.00 | 0 | 0 |
| Dasatinib | 0.41 | 1.00 | 0.97 | 0 | 0 |
| Deferasirox | 0.81 | 1.00 | 0.97 | 0 | 0 |
| Desvenlafaxine | 0.06 | 1.00 | 1.00 | 0 | 0 |
| Dofetilide | 0.00 | 1.00 | 1.00 | 1 | 1 |
| Doripenem | 0.85 | 0.99 | 0.64 | 0 | 0 |
| Duloxetine | 0.05 | 1.00 | 0.83 | 0 | 0 |
| Eltrombopag | 0.00 | 1.00 | 1.00 | 0 | 1 |
| Etravirine | 0.98 | 1.00 | 1.00 | 0 | 0 |
| Everolimus | 0.88 | 0.98 | 1.00 | 0 | 0 |
| Lacosamide | 0.39 | 0.99 | 0.84 | 0 | 0 |
| Lamotrigine | 0.01 | 1.00 | 0.98 | 0 | 1 |
| Lapatinib | 0.02 | 1.00 | 0.98 | 1 | 1 |
| Maraviroc | 0.09 | 1.00 | 1.00 | 0 | 0 |
| Moxifloxacin | 0.03 | 0.95 | 1.00 | 1 | 1 |
| Nebivolol | 0.24 | 0.99 | 1.00 | 0 | 0 |
| Nelfinavir | 0.12 | 1.00 | 1.00 | 1 | 0 |
| Nilotinib | 0.00 | 1.00 | 0.97 | 1 | 1 |
| Paliperidone | 0.02 | 1.00 | 1.00 | 1 | 1 |
| Palonosetron | 0.24 | 0.99 | 0.95 | 0 | 0 |
| Raltegravir | 0.33 | 1.00 | 1.00 | 0 | 0 |
| Sildenafil | 0.19 | 1.00 | 0.91 | 1 | 0 |
| Silodosin | 0.38 | 1.00 | 0.98 | 0 | 0 |
| Sitagliptin | 0.02 | 1.00 | 1.00 | 1 | 1 |
| Solifenacin | 0.04 | 1.00 | 0.95 | 1 | 0 |
| Sunitinib | 0.03 | 1.00 | 1.00 | 1 | 1 |
| Tadalafil | 0.42 | 0.96 | 1.00 | 0 | 0 |
| Telbivudine | 0.87 | 0.98 | 1.00 | 0 | 0 |
| Tolterodine | 0.01 | 1.00 | 1.00 | 1 | 1 |
| Vardenafil | 0.77 | 0.99 | 0.89 | 1 | 0 |

Dog data-set

The Response column in this data-set corresponds to whether a compound causes prolongation (1), no effect (0) or shortening (-1). Only 15 of the compounds were de-anonymised, the rest were anonymised in the original article.

| \| Compound \| SKr \| Sna \| SCaL \| Response \| LOOCV \| \| --- \| --- \| --- \| --- \| --- \| --- \| \| 4-Aminopyridine \| 1.00 \| 1.00 \| 1.65 \| 0 \| 0 \| \| Almokalant \| 0.65 \| 1.00 \| 1.00 \| 1 \| 1 \| \| Amiodarone \| 0.03 \| 0.11 \| 1.04 \| -1 \| -1 \| \| Cisapride \| 0.07 \| 0.31 \| 1.00 \| 0 \| 0 \| \| E-4031 \| 0.05 \| 0.97 \| 1.00 \| 1 \| 1 \| \| FPL-64176 \| 1.00 \| 1.00 \| 1.09 \| 1 \| 0 \| \| Isradipine \| 0.97 \| 0.92 \| 0.01 \| -1 \| -1 \| \| Lidocaine \| 0.92 \| 0.71 \| 1.00 \| -1 \| -1 \| \| Nifedipine \| 1.00 \| 0.90 \| 0.00 \| -1 \| -1 \| \| Pimozide \| 0.31 \| 0.62 \| 0.40 \| -1 \| 0 \| \| Pinacidil \| 0.90 \| 1.00 \| 1.00 \| -1 \| 0 \| \| Quinidine \| 0.05 \| 0.24 \| 0.34 \| 0 \| 0 \| \| Terfenadine \| 0.04 \| 0.12 \| 0.15 \| -1 \| -1 \| \| TTX \| 1.00 \| 0.02 \| 1.00 \| -1 \| -1 \| \| Ziprasidone \| 0.04 \| 0.88 \| 1.00 \| 1 \| 1 \| \| AZ1 \| 1.00 \| 1.00 \| 0.95 \| 1 \| 0 \| \| AZ2 \| 0.48 \| 1.00 \| 1.00 \| 1 \| 1 \| \| AZ3 \| 0.05 \| 1.00 \| 1.00 \| 0 \| 1 \| \| AZ4 \| 0.33 \| 1.00 \| 0.87 \| 1 \| 1 \| \| AZ5 \| 1.00 \| 1.00 \| 0.75 \| 1 \| 0 \| \| AZ6 \| 1.00 \| 1.00 \| 0.56 \| 1 \| 0 \| \| AZ7 \| 1.74 \| 1.00 \| 1.44 \| -1 \| -1 \| \| AZ8 \| 0.53 \| 0.52 \| 0.89 \| -1 \| -1 \| \| AZ9 \| 0.07 \| 1.00 \| 1.00 \| 1 \| 1 \| \| AZ10 \| 0.32 \| 0.33 \| 1.00 \| -1 \| -1 \| \| AZ11 \| 0.15 \| 0.12 \| 1.00 \| -1 \| -1 \| \| AZ12 \| 0.22 \| 1.00 \| 1.00 \| -1 \| 1 \| \| AZ13 \| 1.00 \| 0.68 \| 0.08 \| 0 \| -1 \| \| AZ14 \| 0.14 \| 1.00 \| 1.00 \| -1 \| 1 \| \| AZ15 \| 0.55 \| 0.15 \| 0.95 \| 0 \| -1 \| \| AZ16 \| 0.59 \| 1.00 \| 1.00 \| 1 \| 1 \| \| AZ17 \| 0.10 \| 0.07 \| 0.22 \| -1 \| -1 \| \| AZ18 \| 1.00 \| 1.00 \| 1.00 \| 0 \| 0 \| \| AZ19 \| 0.13 \| 0.25 \| 0.49 \| -1 \| -1 \| \| AZ20 \| 0.13 \| 0.77 \| 0.76 \| 1 \| 1 \| \| AZ21 \| 0.79 \| 1.00 \| 1.00 \| 0 \| 0 \| \| AZ22 \| 1.00 \| 0.94 \| 1.00 \| 0 \| -1 \| \| AZ23 \| 0.79 \| 1.00 \| 1.00 \| 0 \| 0 \| \| AZ24 \| 0.68 \| 0.80 \| 1.00 \| 0 \| 0 \| \| AZ25 \| 0.44 \| 1.00 \| 1.00 \| 0 \| 1 \| \| AZ26 \| 1.00 \| 1.00 \| 0.08 \| -1 \| -1 \| \| AZ27 \| 1.00 \| 1.00 \| 0.10 \| 0 \| -1 \| \| AZ28 \| 0.60 \| 0.82 \| 0.90 \| -1 \| 0 \| \| AZ29 \| 1.08 \| 1.00 \| 1.00 \| -1 \| -1 \| \| AZ30 \| 0.81 \| 1.00 \| 1.00 \| 1 \| 0 \| \| AZ31 \| 0.13 \| 1.00 \| 1.00 \| 1 \| 1 \| \| AZ32 \| 0.28 \| 1.00 \| 1.00 \| 1 \| 1 \| \| AZ33 \| 0.49 \| 0.66 \| 0.27 \| 0 \| 0 \| \| AZ34 \| 0.29 \| 1.00 \| 0.16 \| 0 \| 1 \| \| AZ35 \| 0.09 \| 0.76 \| 1.00 \| -1 \| 1 \| \| AZ36 \| 0.07 \| 0.31 \| 0.91 \| -1 \| 0 \| \| AZ37 \| 0.63 \| 0.57 \| 1.00 \| 0 \| -1 \| \| AZ38 \| 1.00 \| 1.00 \| 1.00 \| 0 \| 0 \| |  |  |  |
| --- | --- | --- | --- | --- | --- | --- | --- | --- | --- | --- | --- | --- | --- | --- | --- | --- | --- | --- | --- | --- | --- | --- | --- | --- | --- | --- | --- | --- | --- | --- | --- | --- | --- | --- | --- | --- | --- | --- | --- | --- | --- | --- | --- | --- | --- | --- | --- | --- | --- | --- | --- | --- | --- | --- | --- | --- | --- | --- | --- | --- | --- | --- | --- | --- | --- | --- | --- | --- | --- | --- | --- | --- | --- | --- | --- | --- | --- | --- | --- | --- | --- | --- | --- | --- | --- | --- | --- | --- | --- | --- | --- | --- | --- | --- | --- | --- | --- | --- | --- | --- | --- | --- | --- | --- | --- | --- | --- | --- | --- | --- | --- | --- | --- | --- | --- | --- | --- | --- | --- | --- | --- | --- | --- | --- | --- | --- | --- | --- | --- | --- | --- | --- | --- | --- | --- | --- | --- | --- | --- | --- | --- | --- | --- | --- | --- | --- | --- | --- | --- | --- | --- | --- | --- | --- | --- | --- | --- | --- | --- | --- | --- | --- | --- | --- | --- | --- | --- | --- | --- | --- | --- | --- | --- | --- | --- | --- | --- | --- | --- | --- | --- | --- | --- | --- | --- | --- | --- | --- | --- | --- | --- | --- | --- | --- | --- | --- | --- | --- | --- | --- | --- | --- | --- | --- | --- | --- | --- | --- | --- | --- | --- | --- | --- | --- | --- | --- | --- | --- | --- | --- | --- | --- | --- | --- | --- | --- | --- | --- | --- | --- | --- | --- | --- | --- | --- | --- | --- | --- | --- | --- | --- | --- | --- | --- | --- | --- | --- | --- | --- | --- | --- | --- | --- | --- | --- | --- | --- | --- | --- | --- | --- | --- | --- | --- | --- | --- | --- | --- | --- | --- | --- | --- | --- | --- | --- | --- | --- | --- | --- | --- | --- | --- | --- | --- | --- | --- | --- | --- | --- | --- | --- | --- | --- | --- | --- | --- | --- | --- | --- | --- | --- | --- | --- | --- | --- | --- | --- | --- | --- | --- | --- | --- | --- | --- | --- | --- | --- | --- | --- | --- | --- | --- | --- | --- | --- | --- | --- |
|  |  |  |  |

Rabbit data-set

The Response column in this data-set corresponds to whether a compound causes prolongation (1), no effect (0) or shortening (-1). All the compounds within this data-set were anonymised in the original article.

| SKr | Sna | SCaL | Response | LOOCV |
| --- | --- | --- | --- | --- |
| 0.54 | 0.96 | 0.92 | 1 | 1 |
| 0.32 | 0.35 | 0.44 | 0 | 0 |
| 0.91 | 0.91 | 0.48 | -1 | -1 |
| 0.92 | 0.98 | 1.00 | 0 | 0 |
| 0.39 | 0.98 | 0.97 | 1 | 1 |
| 0.97 | 0.92 | 0.89 | -1 | -1 |
| 0.29 | 0.94 | 0.88 | 0 | 1 |
| 0.54 | 0.94 | 0.89 | -1 | 1 |
| 0.11 | 0.98 | 0.97 | 1 | 1 |
| 0.95 | 0.99 | 1.00 | 0 | 0 |
| 0.91 | 0.98 | 0.98 | 0 | 0 |
| 0.16 | 0.88 | 0.44 | 1 | 1 |
| 0.60 | 1.00 | 1.00 | 0 | 1 |
| 0.89 | 0.98 | 0.97 | 0 | 0 |
| 0.50 | 0.71 | 0.97 | 0 | 1 |
| 0.91 | 1.00 | 0.77 | -1 | -1 |
| 0.19 | 0.22 | 0.82 | 0 | 1 |
| 0.37 | 0.82 | 0.30 | 1 | 0 |
| 0.99 | 0.99 | 0.97 | 0 | -1 |
| 0.99 | 0.99 | 0.99 | 0 | 0 |
| 0.59 | 0.95 | 0.92 | 1 | 1 |
| 0.77 | 0.53 | 0.87 | 0 | -1 |
| 0.95 | 0.96 | 0.92 | 0 | -1 |
| 0.78 | 0.86 | 0.86 | -1 | 0 |
| 0.81 | 0.72 | 0.89 | 0 | -1 |
| 0.98 | 0.96 | 0.97 | -1 | -1 |
| 0.73 | 0.99 | 0.99 | 1 | 1 |
| 0.43 | 0.79 | 0.92 | 0 | 1 |
| 0.48 | 0.95 | 0.90 | 0 | 1 |
| 0.19 | 0.99 | 0.98 | 1 | 1 |
| 0.91 | 0.99 | 0.97 | 0 | -1 |
| 0.57 | 0.88 | 0.88 | 0 | 1 |
| 0.18 | 0.86 | 0.68 | 1 | 1 |
| 0.18 | 0.97 | 0.98 | 1 | 1 |
| 0.97 | 1.00 | 0.99 | 0 | 0 |
| 0.68 | 0.70 | 0.50 | -1 | -1 |
| 0.79 | 0.98 | 1.00 | 0 | 0 |
| 0.29 | 0.78 | 0.77 | 1 | 1 |
| 0.91 | 0.16 | 0.50 | 0 | -1 |
| 0.68 | 0.99 | 0.95 | 1 | 1 |
| 0.96 | 0.94 | 0.99 | 1 | 0 |
| 0.73 | 0.81 | 0.76 | -1 | 0 |
| 0.88 | 0.98 | 0.96 | 0 | -1 |
| 0.61 | 1.00 | 0.97 | 1 | 1 |
| 1.00 | 1.00 | 0.99 | -1 | 0 |
| 0.32 | 0.62 | 0.63 | 1 | 1 |
| 0.44 | 0.71 | 0.19 | -1 | -1 |
| 0.75 | 0.95 | 0.97 | -1 | 0 |
| 0.58 | 0.90 | 0.93 | 1 | 1 |
| 0.83 | 0.98 | 0.97 | 0 | 0 |
| 0.26 | 0.34 | 0.61 | 1 | 1 |
| 0.97 | 0.99 | 0.98 | 0 | 0 |
| 0.53 | 0.99 | 0.96 | 1 | 1 |
| 0.65 | 0.97 | 0.96 | 1 | 1 |
| 0.85 | 0.96 | 0.89 | 0 | -1 |
| 0.73 | 0.39 | 0.86 | -1 | -1 |
| 0.86 | 0.50 | 0.18 | -1 | -1 |
| 0.87 | 0.96 | 0.87 | 0 | 0 |
| 0.46 | 0.96 | 0.96 | 0 | 1 |
| 0.93 | 1.00 | 0.98 | 0 | 0 |
| 0.29 | 0.87 | 0.77 | 1 | 1 |
| 0.65 | 0.99 | 0.95 | 0 | 1 |
| 0.71 | 1.00 | 0.98 | 0 | 1 |
| 0.22 | 0.46 | 0.55 | 1 | 1 |
| 0.78 | 0.98 | 0.99 | 0 | 0 |
| 0.31 | 0.91 | 0.99 | -1 | 1 |
| 0.68 | 0.99 | 0.99 | 1 | 1 |
| 0.97 | 0.73 | 0.89 | 0 | -1 |
| 0.23 | 0.96 | 0.97 | 1 | 1 |
| 0.14 | 0.57 | 0.63 | 1 | 1 |
| 0.59 | 0.92 | 0.89 | 0 | 1 |
| 0.46 | 0.98 | 0.96 | 1 | 1 |
| 0.95 | 0.97 | 0.77 | 0 | -1 |
| 0.47 | 0.99 | 0.98 | -1 | 1 |
| 0.92 | 0.99 | 0.66 | 0 | -1 |
| 0.86 | 0.98 | 1.00 | -1 | 0 |
| 0.67 | 0.17 | 0.67 | 0 | -1 |

1. **Parameter Values & Final Algorithm**

The classifier Z was defined as:

$$Z=\frac{1+a_{0}S_{CaL}+a_{1}S_{Na}}{1+a_{2}S_{Kr}}$$

This classifier was manipulated via a logistic regression model:

$$Y=\frac{\exp(Z)}{1+\exp(Z)}$$

For the LOOCV, each data-set was associated to a unique set of parameters which was obtained following global fitting to the data. Optimized parameters and cutoff values for Y were as follows:

**Human 1**

a0 = 0.4560, a1 = 0.2075, a2 = 1.0809

Does a drug carry a Torsades de Pointes risk?

If *Y*<= 0.6902 then

No

else

Yes

end

**Human 2**

a0 = 7.5126, a1 = 3.4932, a2 = 4.932

Does a drug carry a Torsades de Pointes risk?

If *Y* <= 0.8865 then

No

else

Yes

end

**Human 3**

a0 = 0.3649, a1 = 0.7596, a2 = 0.4559

Does a drug cause QTc prolongation?

If *Y* <= 0.8888 then

No

else

Yes

end

**Dog**

a0 = 3.22, a1 = 0.3305, a2 = 1.6898

Does a drug cause a >10% prolongation, >10% shortening or have no effect?

If *Y* <= 0.8353 then

Drug = >10% Shortening

else if *Y* > 0.8353 & *Y* <= 0.8908 then

Drug = No Effect

else

Drug = >10% Prolongation

end

end

**Rabbit**

a0 = 5.6454, a1 = 6.6867, a2 = 9.3254

Does a drug cause a >10% prolongation, >10% shortening or have no effect?

If *Y* <= 0.7836 then

Drug = >10% Shortening

else if *Y* > 0.7836 & *Y* <= 0.844 then

Drug = No Effect

else

Drug = >10% Prolongation

end

end
